# Perinatal Azithromycin Provides Limited Neuroprotection in an Ovine Model of Neonatal Hypoxic-Ischemic Encephalopathy

**DOI:** 10.1161/STROKEAHA.123.043040

**Published:** 2023-10-17

**Authors:** Jana Krystofova Mike, Yasmine White, Rachel S. Hutchings, Christian Vento, Janica Ha, Hadiya Manzoor, Donald Lee, Courtney Losser, Kimberly Arellano, Oona Vanhatalo, Elena Seifert, Anya Gunewardena, Bo Wen, Lu Wang, Aijun Wang, Brian D. Goudy, Payam Vali, Satyan Lakshminrusimha, Jogarao V.S. Gobburu, Janel Long-Boyle, Yvonne W. Wu, Jeffrey R. Fineman, Donna M. Ferriero, Emin Maltepe

**Affiliations:** Department of Pediatrics (J.K.M., Y.W., R.S.H., C.V., J.H., C.L., K.A., O.V., E.S., A.G., J.L.-B., Y.W.W., J.R.F., D.M.F., E.M.), University of California San Francisco.; School of Pharmacy (J.L.-B.), University of California San Francisco.; Department of Neurology, Weill Institute for Neurosciences (Y.W.W., D.M.F.), University of California San Francisco.; Department of Biomedical Sciences (E.M.), University of California San Francisco.; School of Pharmacy, University of Maryland, Baltimore (D.L., J.V.S.G.).; College of Pharmacy, University of Michigan, Ann Arbor (B.W., L.W.).; Department of Biomedical Engineering (H.M., A.W.), University of California Davis.; Department of Pediatrics (B.D.G., P.V., B.D.G., P.V., S.L., J.-L.B., O.V.), University of California Davis.; Initiative for Pediatric Drug and Device Development, San Francisco, CA (J.V.S.G., J.R.F., E.M.).

**Keywords:** asphyxia, azithromycin, brain hypoxia-ischemia, neonates, ovine model

## Abstract

**BACKGROUND:**

Hypoxic-ischemic brain injury/encephalopathy affects about 1.15 million neonates per year, 96% of whom are born in low- and middle-income countries. Therapeutic hypothermia is not effective in this setting, possibly because injury occurs significantly before birth. Here, we studied the pharmacokinetics, safety, and efficacy of perinatal azithromycin administration in near-term lambs following global ischemic injury to support earlier treatment approaches.

**METHODS:**

Ewes and their lambs of both sexes (n=34, 141–143 days) were randomly assigned to receive azithromycin or placebo before delivery as well as postnatally. Lambs were subjected to severe global hypoxia-ischemia utilizing an acute umbilical cord occlusion model. Outcomes were assessed over a 6-day period.

**RESULTS:**

While maternal azithromycin exhibited relatively low placental transfer, azithromycin-treated lambs recovered spontaneous circulation faster following the initiation of cardiopulmonary resuscitation and were extubated sooner. Additionally, peri- and postnatal azithromycin administration was well tolerated, demonstrating a 77-hour plasma elimination half-life, as well as significant accumulation in the brain and other tissues. Azithromycin administration resulted in a systemic immunomodulatory effect, demonstrated by reductions in proinflammatory IL-6 (interleukin-6) levels. Treated lambs exhibited a trend toward improved neurodevelopmental outcomes while histological analysis revealed that azithromycin supported white matter preservation and attenuated inflammation in the cingulate and parasagittal cortex.

**CONCLUSIONS:**

Perinatal azithromycin administration enhances neonatal resuscitation, attenuates neuroinflammation, and supports limited improvement of select histological outcomes in an ovine model of hypoxic-ischemic brain injury/encephalopathy.

While hypoxic-ischemic brain injury/encephalopathy (HIE) is the leading cause of neonatal morbidity and mortality worldwide (≈4 million neonates annually) and accounts for nearly 25% of neonatal deaths (≈1 million newborns), low- and middle-income countries (LMICs) carry the largest burden with 10× the numbers observed in high-resource settings.^1^ Multiple factors impact the incidence and mortality of HIE in neonates in LMIC, including limited preventive antepartum and intrapartum care, as well as, critical care resources.^2^ One million infants per year survive with cerebral palsy and serious developmental disabilities.^3–5^ While therapeutic hypothermia is standard of care and can reduce brain injury and mortality following birth asphyxia in high-income countries,^6^ this was not observed in a large trial in the LMIC setting^7^ where a lower incidence of sentinel events and higher seizure burden at randomization suggest an in utero injury evolving earlier over the course of labor.^8^ Therapeutic agents that could be used prophylactically during labor, as well as immediately postnatally, may therefore be necessary to improve outcomes globally.

One potential candidate is azithromycin. In addition to its known antibiotic effects, azithromycin possesses immunomodulatory properties via its ability to modulate inflammation, reactive oxygen species formation, cell senescence, and phagocytosis.^9^ Azithromycin’s effects on nonimmune cells, such as endothelial or epithelial cells, as well as fibroblasts can further help maintain integrity of the blood-brain barrier.^9^ Collectively, these effects can translate into protective effects in animal models of HIE, where azithromycin has been studied in cerebral artery occlusion in mice, inflammation-amplified or isolated right carotid artery ligation in rats, as well as models of spinal cord injury.^10–13^

Importantly, azithromycin is widely available, easy to store and administer, and well studied in the pediatric and obstetric populations, making it a promising therapeutic candidate for use in the perinatal setting. Recently, large-scale biannual prophylactic azithromycin administration was trialed in some sub-Saharan African settings where biannual mass azithromycin administration reduced all-cause mortality in children aged 1 month to 5 years.^14^ Similarly, azithromycin administration prophylactically during labor induction of nulliparous patients with obesity was well tolerated and significantly reduced cesarean delivery as well as maternal morbidity.^15–18^ We therefore evaluated the pharmacokinetics, safety, and neuroprotective effects of perinatal azithromycin administration in our clinically relevant delivery model of HIE in near-term lambs designed to develop therapies for LMIC.

## METHODS

### Data Availability

The data presented in this work are available upon reasonable request. Full descriptions of Methods are available in the Supplemental Material.

### Animals

All animal research was approved by the University of California Davis Institutional Animal Care and Use Committee and was performed in accordance with the Guide for the Care and Use of Laboratory Animals. ARRIVE guidelines were additionally utilized.^19^

### Neonatal Hypoxia-Ischemia

Sheep of both sexes were used. HIE was induced via umbilical cord occlusion (UCO) in near term lambs at 141 to 143 days gestation (term ~ 147–150 days). Details are described in Supplemental Material.

### Drug Treatment

**Figure 1.**
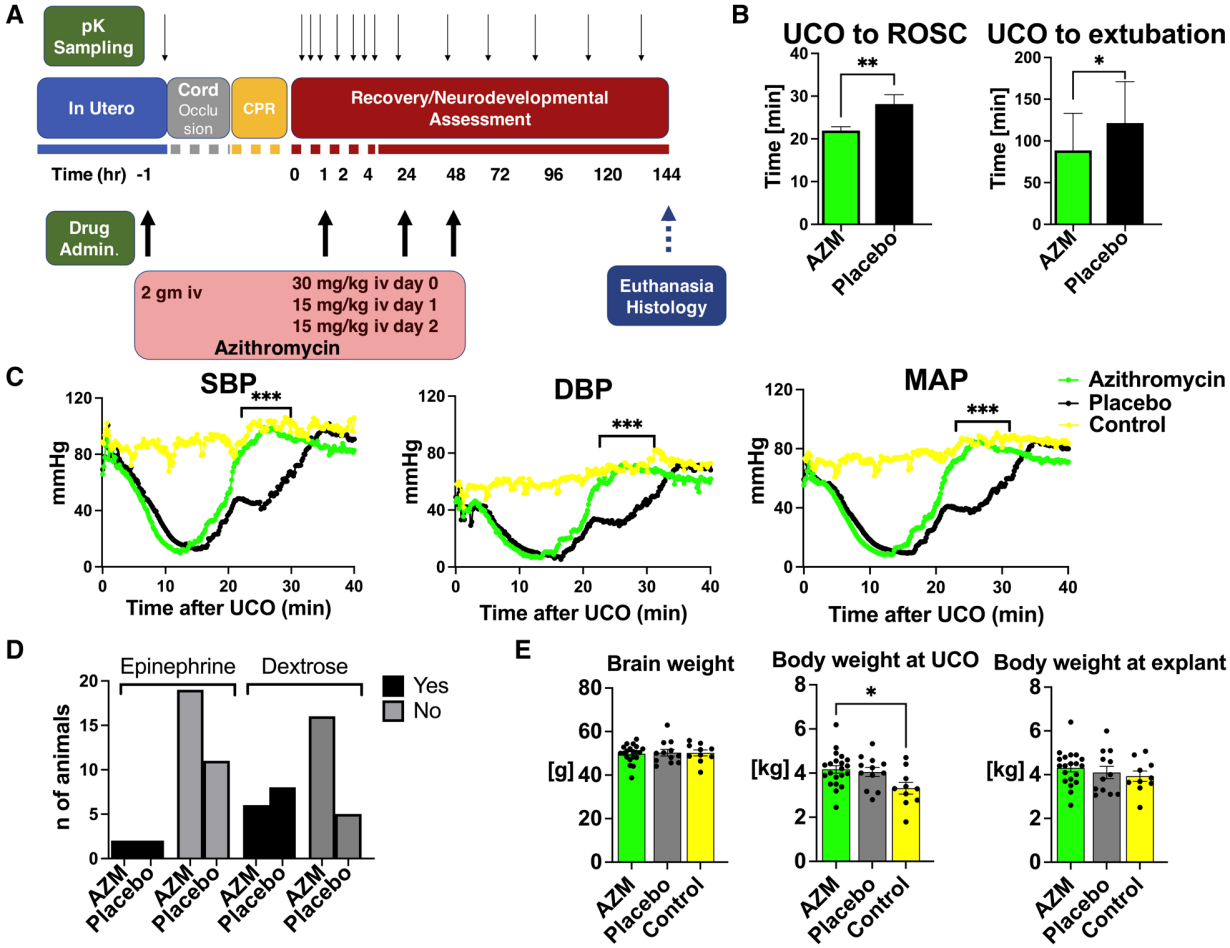
**Umbilical cord occlusion (UCO) model and resuscitation outcomes. A**, Timeline of azithromycin (AZM) administration. **B**, Return of spontaneous circulation (ROSC) was faster in AZM-treated animals, and the time to extubation was shorter compared with the placebo group. Groups were compared using unpaired *t* test for ROSC assessment and Mann-Whitney *U* test for extubation time. AZM: n=21, placebo: n=13. **C**, UCO (time 0 min) induced asystole with corresponding loss in cardiac output and hypotension. ROSC restored blood pressure to levels similar to age-matched controls. Hemodynamic data were analyzed using grouped analysis of the individual group’s means for a specific time point. AZM: n=18; placebo: n=13; control: n=6 (controls data were used with permission from Mike et al^23^). **D**, The incidence of second dose of epinephrine and dextrose administration was similar between the groups. The proportion of variables was assessed using Fisher exact test. AZM: n=21; placebo: n=13. **E**, There were no differences in selected anthropometric parameters between AZM and placebo. Brain and body weight differences were assessed using ANOVA. AZM: n=21; placebo: n=12; control: n=10. Data shown in the graphs **B** and **E** as mean±SEM, graph **C** are shown as mean, graph **D** is a contingency table. AZM-treated group, green. Placebo, black. Controls, yellow. CPR indicates cardiopulmonary resuscitation; DBP, diastolic blood pressure; MAP, mean arterial pressure; pK, pharmacokinetic; and SBP, systolic blood pressure. *P<0.05, **P<0.01, ***P<0.001.

Azithromycin dose was defined by a pilot dose-finding pharmacokinetic study (see Supplemental Material). In the randomized efficacy study, treatment consisted of 2 g IV azithromycin administered over 1 hour to the ewe before delivery and 30 mg/kg IV azithromycin administered to the lamb as a one-hour IV infusion starting 10 minutes after resuscitation followed by 2 additional 15 mg/kg IV azithromycin doses at 24 and 48 hours of life (Figure 1A).

### Pharmacokinetic Analysis

Azithromycin levels in ewe and lamb plasma and tissue samples were quantified using liquid chromatography with tandem mass spectrometry (see Supplemental Material). Plasma pharmacokinetic parameters for azithromycin were estimated utilizing standard noncompartmental analysis with R package NonCompart (Version 0.6.0; Bae, 2022) in R version 4.2.0 and with PumasAI/NCA.jl (Version 1.2.10; Pumas-AI Inc, Baltimore, MD) followed by nonlinear mixed effect modeling using Pumas (Version 1.1; Pumas-AI Inc, Baltimore, MD).^20^

### Neurological Outcomes Assessments

Neurological outcomes were evaluated daily for 6 days. Our neurobehavioral assessment was based on observations previously conducted in sheep to monitor their well being following birth^21,22^ and included evaluation of motor function, feeding, and activity at rest. See Supplemental Material for additional details.

### Neurohistopathology and Image Analysis

Structural and inflammatory changes at 6 days after UCO were measured using qualitative and morphological assessment of gray and white matter structures in a blinded manner, as described previously.^23^ Analysis included the average of 3 images from one anatomic area of interest. We assessed 3 regions of white matter represented by periventricular white matter (PVWM), subcortical white matter of the cingulate and first parasagittal gyrus, and 6 regions of gray matter represented by cortex of the cingulate and first parasagittal gyrus (Ctx1 and Ctx2), caudate, putamen (Put), and hippocampal areas (Ca1/2 and Ca3). For details, see Supplemental Material.

### Biochemical Markers of Inflammation

Systemic inflammation was assessed by measuring cytokine levels in serum samples 6 days after the UCO. We also assessed the differences in peripheral blood cell components and peripheral blood cell ratios at baseline before UCO (pre-UCO), at 8 hours of life, and on days 1, 2, 3, 5, and 6 (see Supplemental Material).

### Statistical Analysis

Analyses of biochemical, hemodynamics and histological data were performed using Prism 9 (Version 9.4.1; GraphPad Software, San Diego, CA). The comprehensive description is provided in the Supplemental Material.

## RESULTS

### Azithromycin Pharmacokinetics

Mean placental transfer was 1.6% (range, 0.5%–4.1%) in 6 sets of paired ewe and lamb plasma samples collected immediately before UCO and delivery. Individual lamb plasma azithromycin concentration-time plots are shown in Figure S1A. Noncompartmental analysis of this cohort indicated median C_max_, 4.1 mg/L (range, 2.3–18.0 mg/L); median AUC_inf_, 154.6 mg×h/L (range, 110.3–224.9 mg×h/L); and median terminal elimination half-life, 77.3 hours (range, 35.6–137.7 hours). Population pharmacokinetic modeling showed that a 2-compartment model with weight as a covariate on clearance best fit the data. Parameter estimates for the final model including proportional error are listed in Table S1. Individual model predicted concentrations (Figure S1B), standard goodness-of-fit plots (Figure S1C and S1D), and the visual predictive check (Figure S1E) demonstrates the final model described the observed data without bias.

Azithromycin concentrations on day 6 in all tissues examined were much higher than in concurrent plasma samples (Table). To maximize azithromycin exposure, a dosing regimen of 2 g IV azithromycin was administered to the ewe 1 hour before delivery followed by 30 mg/kg IV azithromycin administered to the lamb following resuscitation and 15 mg/kg IV azithromycin administered at 24 and 48 hours of life was selected for the randomized efficacy study.

**Table.**
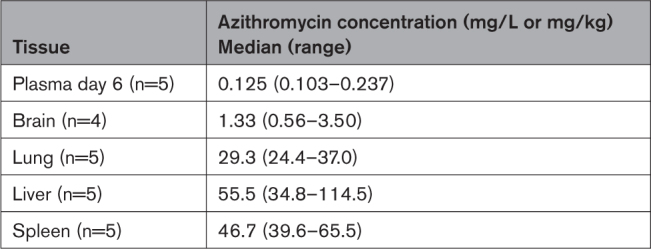
Tissue Concentrations of Azithromycin in Neonatal Lambs at 6 Days of Life

### Physiological Outcomes

Azithromycin-treated animals demonstrated earlier return of spontaneous circulation (ROSC) following CPR and were able to be extubated sooner than their placebo counterparts (Figure 1B) suggesting that perinatal azithromycin administration may enhance hemodynamic response to CPR (Figure 1C). Epinephrine was administered to all animals in both groups. Most animals required one dose and 2 animals from each group required >2 doses (*P*=0.62). Hypoglycemia defined as glucose <50 mg/mL during recovery before initiation of feeds was detected in 6 animals in the azithromycin-treated and 7 animals in the placebo group (*P*=0.13; Figure 1D). azithromycin administration relative to placebo did not lead to a change in brain weight, body weight at UCO, or body weight at explant (Figure 1E).

### Biochemical Outcomes

Serial arterial blood gasses were drawn at baseline preUCO, immediately before initiation of CPR and at 10, 20, 30, and 60 minutes after ROSC. Consistent with our prior studies,^23^ the UCO protocol produced a clinically significant combined metabolic and respiratory acidosis (Table S3, 1A). Azithromycin-treated lambs were slightly more acidic at the end of asphyxia just before CPR compared with the placebo group as evidenced by lower pH values (*P*=0.006). However, no differences in lactate levels or base excess were observed between groups at any of the time points studied. There were no significant changes in oxygenation and ventilation among the studied groups. compared with controls (controls data were used with permission from Mike et al^23^), both azithromycin and placebo group lambs experienced hyperoxia 10 minutes after the CPR due to use of oxygen during the resuscitation (azithromycin, *P*=0.0007; placebo, *P*=0.004) and hyperglycemia (azithromycin, *P*=0.0002; placebo, *P*=0.024) reflecting the stress response to the UCO and epinephrine administration (Table S3, 1A). Azithromycin did not exhibit noticeable toxicity, as reflected by similar chemistry panels relative to placebo-treated animals (Table S3, 1B).

### Inflammation

**Figure 2.**
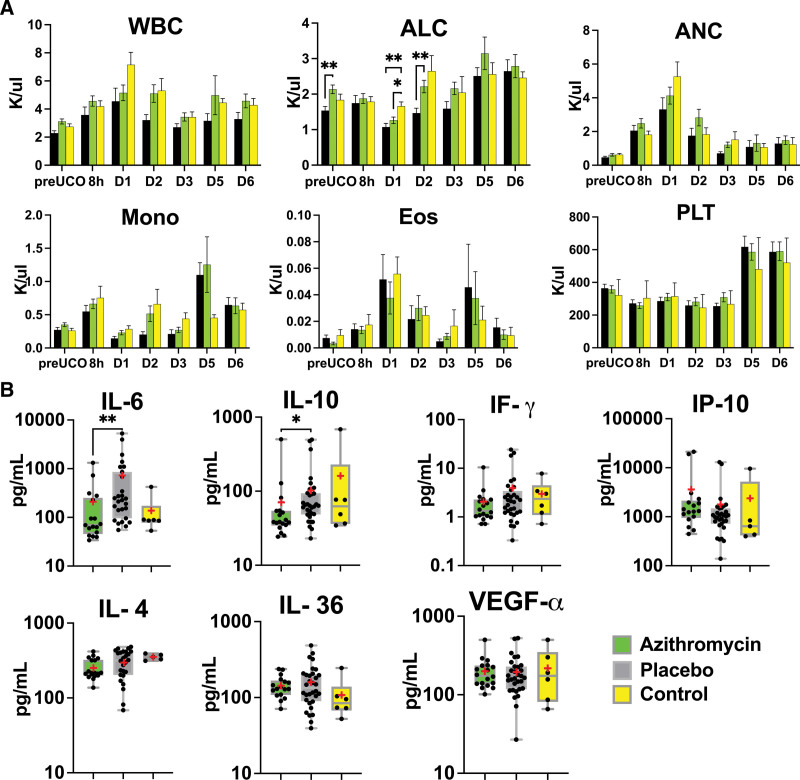
**Peripheral markers of inflammation.** A, Absolute lymphocyte count (ALC) differed at baseline and on days 1 and 2 in azithromycin (AZM) vs placebo group. Cellular subgroups were evaluated by mixed-effect analysis with Smidak correction for multiple comparisons. The summary column graphs show means±SEM. AZM: n=8 to 21; placebo: n=7 to 12; control: n=4 to 21 (control data were used with permission from Mike et al^23^). **B**, At 6 days after the umbilical cord occlusion (UCO), we noticed changes in IL-6 (interleukin-6) and IL-10 (interleukin-10). No changes were observed in IL-4 (interleukin-4), IF-g (interferon-g), IP-10 (interferon-g inducible protein), IL-36 (interleukin-36), or VEGF-α (vascular endothelial growth factor-α). We used Kruskal-Wallis test. AZM: n=15 to 19; placebo: n=27 to 32; control: n=5 to 6. The box graphs show the mean as red asterisk “*” and the median with interquartile range. AZM-treated group, green. Placebo, black. Controls, yellow. ANC indicates absolute neutrophil count; Eos, eosinophil; Mono, monocyte; PLT, platelet; and WBC, white blood cell. *P<0.05, **P<0.01.

Given azithromycin’s known role in modulating the immune system, we assessed basic markers of inflammation including white blood cell counts with differential and select cytokine levels. The azithromycin-treated group exhibited slightly higher absolute lymphocyte count at baseline (*P*=0.003) and on day 2 compared with placebo lambs (*P*=0.007). Both, azithromycin and placebo lambs had lower absolute lymphocyte count on day 1 after the UCO (azithromycin, *P*=0.002; placebo, *P*=0.03). We did not observe changes in other subgroups of peripheral blood cells (Figure 2A), or peripheral blood cell indices (Figure S2) between the azithromycin and placebo groups. Compared to controls, both azithromycin and placebo-treated lambs had higher systemic immune-inflammation index score (azithromycin, *P*=0.02; placebo, *P*=0.01) on day 2, LMR on day 3 (azithromycin, *P*=0.04; placebo, *P*=0.003). LMR was elevated on day 4 in the azithromycin group (*P*=0.03), and neutrophil to lymphocytes ratio was higher on day 2 in the placebo group (0.006; Figure S2).

From selected cytokines, we detected significantly lower levels of IL-6 (interleukin-6; *P*=0.009) and lower levels of IL-10 (interleukin-10; *P*=0.02) in the azithromycin-treated animals (Figure 2B).

### Neurohistopathological Outcomes

#### White Matter Injury

**Figure 3.**
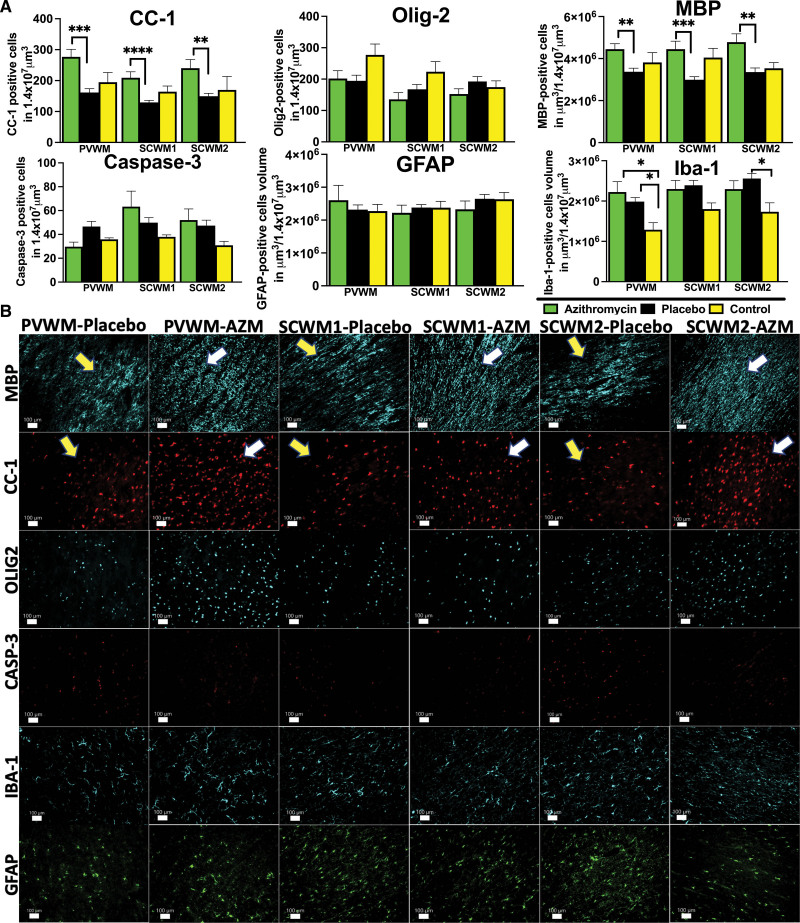
**Quantitative analysis of white matter markers and markers of inflammation.** A, The number of antiadenomatous polyposis coli clone CC-1 (CC-1), and the volume of MBP (myelin basic protein)-stained myelin fibers reflect the changes triggered by umbilical cord occlusion in periventricular white matter (PVWM), subcortical white matter of cingulate gyrus (SCWM1), and subcortical white matter of the first parasagittal gyrus (SCWM2) in azithromycin (AZM)-treated (green) vs placebo-treated (black) vs control (yellow) animals. The cellular death was quantified by the number of cleaved Casp-3 (caspase-3)-positive cells. The neuroinflammation was quantified by the total volume of GFAP (glial fibrillary acidic protein)-positive glial and microglial Iba-1-positive cells. Analyzed were AZM: n=7 to 20, placebo: n=6 to 11, and control: n=5 to 9 animals (control data were used with permission from Mike et al^23^) using either ANOVA or Kruskal-Wallis test. Data are presented as mean±SEM. Brackets show significance as follows: *P<0.05, **P<0.01,***P<0.001, ****P<0.0001. **B**, The representative photomicrographs of histological changes with preservation of the myelin fibers (MBP) and mature oligodendrocytes (CC-1) in AZM-treated group (MBP), myelin breaks, and loss of CC-1 in the placebo group. Cellular death (Casp-3) and gliosis (GFAP) were unchanged among the groups, and more microglial (Iba-1 [ionized calcium-binding adaptor molecule-1]) accumulation was observed in PVWM in both groups and placebo group in SCWM2. Yellow arrows—placebo histopathology, white arrows—AZM-treated histopathology.

White matter injury was assessed by measuring the quantity and integrity of the major structural components of the myelin sheath by anti-MBP (myelin basic protein) immunoreactivity, the quantity of mature oligodendrocytes stained with CC-1, and total oligodendrocyte numbers labeled by Olig-2. The presence of gliosis was detected by the accumulation of GFAP-positive astroglial and Iba-1-positive microglial cells. Azithromycin-treated animals exhibited higher volumes of MBP-positive fibers compared with placebo in all areas studied (PVWM: *P*=0.003; subcortical white matter of cingulate gyrus: *P*=0.0002; subcortical white matter of the first parasagittal gyrus: *P*=0.005) and more mature oligodendrocytes stained with CC-1 (PVWM=0.0003; subcortical white matter of cingulate gyrus: *P*<0.00001; subcortical white matter of the first parasagittal gyrus: *P*=0.0014). The total number of oligodendrocytes did not show significant differences between the studied groups, (Figure 3A and 3B). Azithromycin treatment did not impact cell death or gliosis in white matter regions (Figure 3A and 3B). Increased microglial infiltration was also noticed in PVWM in both treatment groups compared with uninjured controls (azithromycin, *P*=0.01; placebo, *P*=0.03), as well as in the subcortical white matter of the first parasagittal gyrus in the placebo group compared with controls (*P*=0.03; Figure 3A).

#### Gray Matter Injury

**Figure 4.**
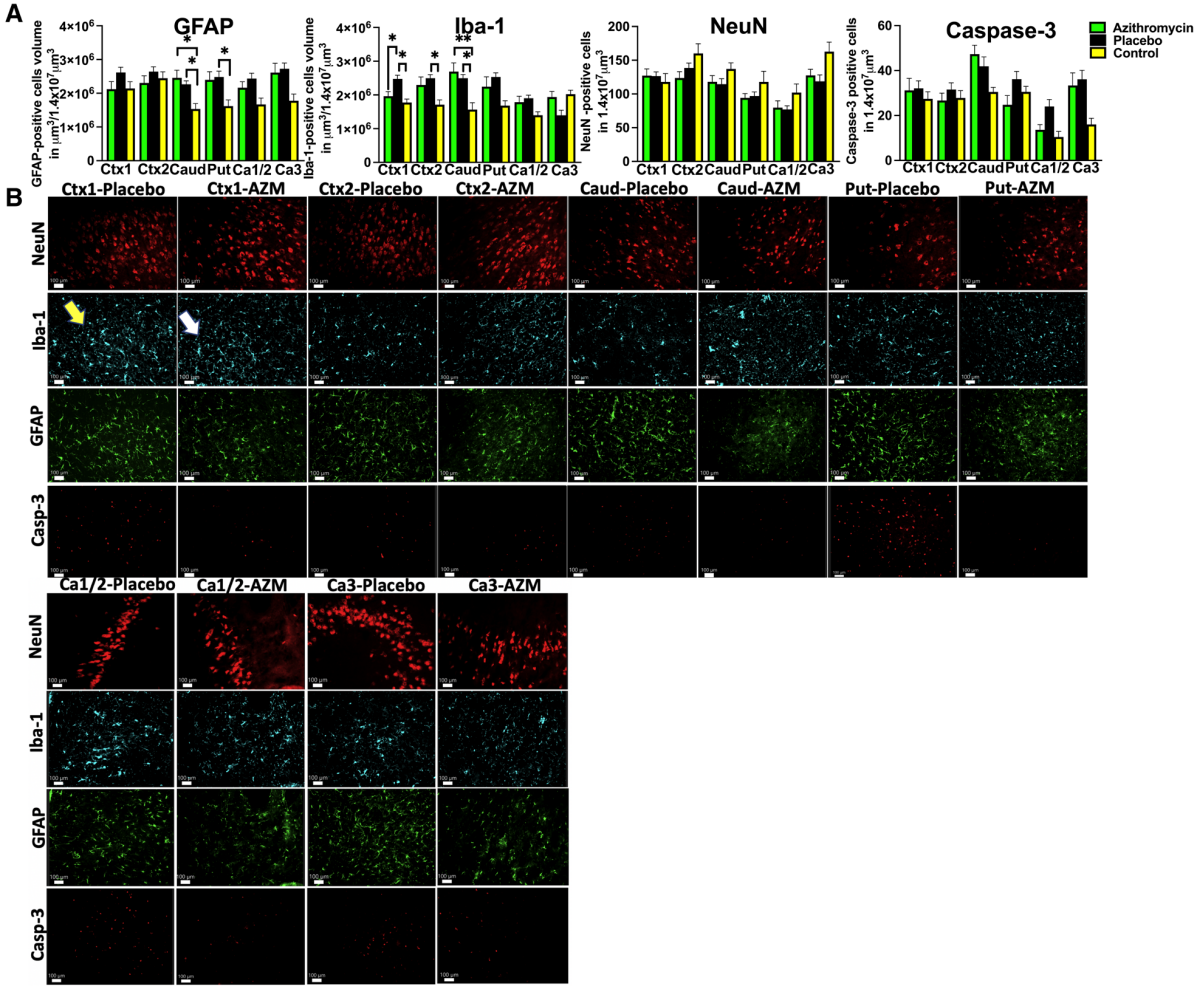
**Histological changes in gray matter. A**, We compared quantitative changes in inflammatory markers of gliosis (GFAP [glial fibrillary acidic protein]) and microglial accumulation (Iba-1 [ionized calcium-binding adaptor molecule-1]); neuronal counts (neuronal nuclei [NeuN]), cellular death markers (Casp-3 [caspase-3]) in cingulate gyrus (Ctx1), first parasagittal gyrus (Ctx2), caudate (Caud), putamen (Put), and Ca1/2 and Ca3 of the hippocampus. Analyzed were azithromycin (AZM): n=7 to 20, placebo: n=6 to 11, controls: n=5 to 8 (control data were used with permission from Mike et al^23^) using either ANOVA or Kruskal-Wallis test. Data are presented as mean±SEM. Brackets show significance as follows: *P<0.05, **P<0.01. AZM-treated animals (green) vs placebo (black) vs control (yellow). **B**, The observed quantitative changes are represented in photomicrographs by accumulation of microglial cells in Ctx-1 (Iba-1 marker), thickened glial cells in Caud in both groups, and Put in the placebo group (GFAP). Placebo animal histologies (yellow arrow) are compared with the AZM-treated animals (white arrow).

Impact of azithromycin treatment on gray matter injury was assessed by evaluating the response of glial cells to injury, neuronal preservation, and quantification of markers of cell death at day 6. Azithromycin treatment prevented microglial accumulation in the cortex of the cingulate (azithromycin versus placebo: *P*=0.01; placebo versus control: *P*=0.02) as well as cortex of the first parasagittal gyrus (placebo versus control: *P*=0.034) and putamen (placebo versus control: *P*=0.04; Figure 4A and 4B). Greater microglial and glial accumulation was noticed in both the placebo and azithromycin-treated groups compared with uninjured controls in caudate (Iba-1: azithromycin, *P*=0.02; placebo, *P*=0.04; GFAP: azithromycin, *P*=0.008; placebo, *P*=0.02; Figure 4A and 4B). Azithromycin treatment did not change the total number of total neuronal cells (Figure 4A and 4B) or caspase-3-positive cells undergoing apoptosis were unchanged in all groups studied in all anatomic areas (Figure 4A and 4B).

### Neurobehavioral Milestones

**Figure 5.**
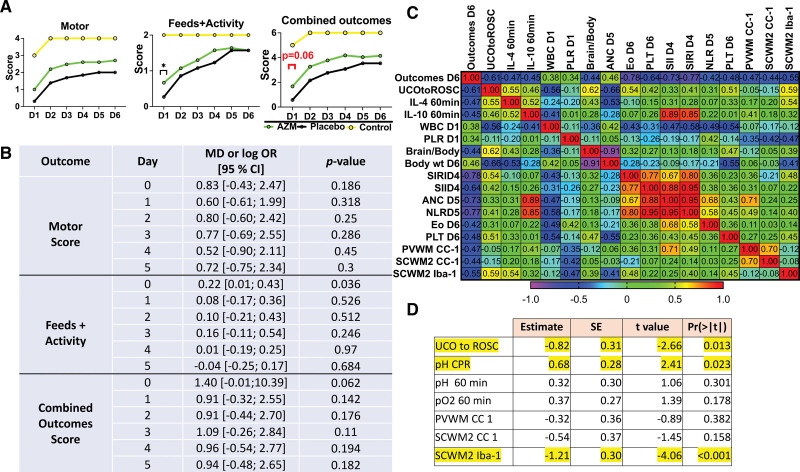
**Neurological outcomes.** A, We assessed combined outcomes score, as a composite score consisting of motor function and feeds+activity. The summary graphs picture the relationship to uninjured controls. Azithromycin (AZM)-treated group, green. Placebo, black. Controls, yellow (control data were used with permission from Mike et al^23^). **B**, Odds ratios of outcomes measures are reported on a logarithmic scale. The statistical approach is described in the Supplemental Material. Data are presented as mean±SEM. Compared were AZM: n=21, placebo: n=13. *P*<0.05 was considered significant. **C**, The correlation matrix pictures Spearman correlation coefficients of selected study parameters with combined neurological outcomes scores on day 6 after the umbilical cord occlusion (UCO) that reached statistical significance (*P*<0.05). The earliest parameters that correlate with neurological outcomes are time from UCO to return of spontaneous circulation (ROSC), total white blood cell (WBC) counts, and platelets to lymphocytes ratio (PLR) cell ratio on day 1. At a later time point, brain-to-body ratio, the body weight at explant, systemic inflammation response index (SIRI), systemic immune-inflammation index (SII) on day 4, absolute neutrophil count (ANC), neutrophil to lymphocytes ratio (NLR) on day 5, eosinophils (Eos), and platelet (PLT) on day 6, and histological markers of mature oligodendrocytes correlate with neurological outcomes on day 6. **D**, Lasso analysis of data revealed UCO to ROSC, pH at CPR, and microgliosis in SCWM2 to predict neurological outcomes on day 6. Yellow: *P*<0.05, n=34. Pre-UCO prior to the umbilical cord occlusion. CC-1 indicates antiadenomatous polyposis coli clone CC-1; CPR, cardiopulmonary resuscitation; Iba-1, ionized calcium-binding adaptor molecule-1; IL-4, interleukin-4; IL-10, interleukin-10; PVWM, periventricular white matter; and SCWM2, subcortical white matter of the first parasagittal gyrus.

We evaluated whether azithromycin treatment produced clinically relevant improvements in neurodevelopmental outcomes in lambs subjected to HIE. Specifically, we assessed motor function, activity at rest, and ability to feed. All injured animals demonstrated significant encephalopathy. Consistent with improvements in acute resuscitation parameters, azithromycin-treated lambs scored higher with respect to feeds and activity, as well as combined outcomes scoring at 8 hours of life (day 0). However, there were trends toward improved motor, activity, and combined outcome score on each day following resuscitation that did not reach statistical significance (Figure 5A and 5B).

### Markers Associated With Neurological Outcomes

Finally, we assessed whether any of the parameters investigated in this study were associated with poor neurological outcomes on day 6 following UCO. From the earliest parameters, worse outcomes correlated with time from UCO to ROSC, white blood cell count, and PLR on day 1. Parameters measured at later time points that correlate with poor neurological outcomes are white blood cell on days 2, 3 (not shown), systemic immune-inflammation index on day 4, systemic inflammation response index on days 4 and 5 (not shown), absolute neutrophil count and neutrophil to lymphocytes ratio on day 5, Eo and platelet on day 6 and brain to body ratio, body weight at explant, and total number of mature oligodendrocytes in PVWM, subcortical white matter of the first parasagittal gyrus, and gliosis in subcortical white matter of the first parasagittal gyrus (Figure 5C). We further assessed whether any of the markers could predict neurological outcomes on day 6. We used Lasso regression, where the lowest mean absolute error occurred with 7 covariates in the model. The model was a good fit with an R^2^ value of 0.71. The top 3 variables that showed statistical significance <0.05 were from early markers including time from UCO to ROSC and pH during CPR and from late assessment of microglial accumulation in the first parasagittal gyrus at day 6 (Figure 5D).

## DISCUSSION

Our results suggest that perinatal azithromycin administration is safe and may provide limited improvement in some outcomes for babies born with HIE in LMIC. Azithromycin accumulates in the placenta and readily crosses the blood-brain barrier.^24,25^ Significant accumulation of azithromycin has previously been reported in fetal tissues following a single IV dose to the pregnant ewe.^24,26^ Consistent with this, while plasma levels of azithromycin in our study were significantly less than those associated with neuroprotection in rodent studies, brain tissue levels were comparable.^27^ Azithromycin has particularly desirable pharmacokinetic properties at sites of ischemic injury as it has increased stability at an acidic pH, a long terminal elimination half-life of 48 to 72 hours, and the ability to achieve high tissue concentrations by delivery via phagocytes,^28^ which likely contribute to azithromycin’s neuroprotective effects more than plasma levels alone.

Immune dysregulation following stroke worsens neurological outcomes.^29^ While azithromycin treatment in our model only altered the immune cell repertoire minimally, cytokine profiles were significantly modulated. A characteristic cytokine gene expression kinetics follows cardiac arrest in adults, as well as in neonates after HIE.^30,31^ At 6 days after the injury, placebo animals demonstrated a persistent proinflammatory state reflected by higher IL-6 levels. In humans, high IL-6 levels correlate with early neurological deterioration, cerebral perfusion deficits, infarct volume, and poor long-term outcomes.^32,33^ We speculate that the increase in IL-10 levels observed in the placebo group may be a compensatory response to counteract the pro-inflammatory state. We additionally observed less evidence of neuroinflammation at the local level. Azithromycin can inhibit oxidative stress-induced morphological changes in glial cells. This observation likely translates to our measurements of lower glial and microglial cell volumes^23,34^ following azithromycin administration. Gliosis increases GFAP expression leading to an increase in tissue stiffness, vascular breakdown, and neuronal cell death.^35^ Azithromycin prevents oligodendrocyte progenitor cell damage, restores differentiation arrest, and potentially myelination ability in an inflammatory environment following exposure to lipopolysaccharide-activated microglia.^36^ In our study, perinatal azithromycin administration appeared to increase total numbers of mature oligodendrocytes and myelin volume in all white matter areas investigated. While significant differences were observed between injured animals that received azithromycin and those that did not, it seems that white matter regions in injured lambs that received azithromycin exhibited a trend toward accentuated development even in comparison to control uninjured animals. Further studies are needed to more clearly define whether azithromycin contributes to oligodendrocyte and myelin formation in the context of the developing ischemic brain, and what mechanisms may be contributory.

An unexpected finding in our study was the improved hemodynamic response to resuscitation in lambs treated with azithromycin. Considering the timing of administration in relation to UCO, the observed effect of azithromycin on ROSC is likely driven by fetal tissue accumulation following maternal administration rather than plasma levels alone. The effects of antenatal azithromycin administration on hemodynamic indices have previously been assessed in nonhuman primates where maternal azithromycin treatment of urea plasma infection improved fetal hemodynamic indices and cardiac function.^37^ A similar cardioprotective mechanism was described early following myocardial infarction.^38^ We additionally speculate that one of the possible mechanisms of improved time to ROSC in the azithromycin-treated group could be via an effect on steroid synthesis. Azithromycin has been shown to enhance corticosterone and cortisol levels^39^ as early as 60 minutes after administration.^40^ Studies investigating this mechanism are ongoing.^41^ Importantly, some studies indicate that steroid administration after cardiac arrest can improve ROSC.^42^ Earlier ROSC has important clinical significance, as it typically is associated with better outcomes.^43^

Finally, we observed slight improvements in neurodevelopmental outcomes only at early time points. Azithromycin-treated animals were less encephalopathic and more vigorous compared with placebo-treated animals at early time points. This probably led to an improved ability to feed after UCO. Feeding difficulties are seen in >50% of severely affected cases, and especially in low-resource settings contributes to malnutrition and poor outcomes.^44^

Potential reasons for the limited neuroprotection observed with azithromycin include limited duration of follow-up. Although 6 days is longer than many large animal studies, it may represent a transitional phase between tissue injury and repair mechanisms in terms of histological observations, and longer observation periods may be needed to fully capture the impact of azithromycin on neurological outcomes. Furthermore, the relatively small sample size limited our power to assess gender differences, an established risk factor for adverse neurological outcomes.

In summary, perinatal azithromycin improves select biochemical, physiological, and histological outcomes in near-term lambs after HIE. While its excellent safety profile positions azithromycin well for use in the perinatal setting, the limited neuroprotective properties demonstrated in our studies suggest it may best be utilized in combination with other agents.

## ARTICLE INFORMATION

### Acknowledgments

The authors thank UCSF Pediatric Critical Care Division.

### Sources of Funding

This work was supported by Bill & Melinda Gates Foundation (Dr Maltepe), NIH grants R35- 5R35NS097299 (Dr Ferriero), R01 HD072455 (Dr Maltepe), K12HD105250 (Dr Mike), K08NS125042 (Dr Mike), T32GM007546 (Dr White), P30CA046592 (Drs Wen and Wang).

### Disclosures

None.

### Supplemental Material

Expanded Methods

Tables S1–S3

Figures S1–S2

ARRIVE guidelines checklist

References 45–47

## Supplementary Material


