# Supplementary material for: Perinatal Azithromycin Provides Limited Neuroprotection in an Ovine Model of Neonatal Hypoxic-Ischemic Encephalopathy
Source: Stroke. 2023 Oct 17;54(11):2864–74. doi: 10.1161/STROKEAHA.123.043040 (PMC10589434; doi:10.1161/STROKEAHA.123.043040)
Supplement: Supplementary file 1 [file str-54-2864-s001.pdf]

Supplemental Material

Perinatal azithromycin provides limited neuroprotection in an ovine model of neonatal hypoxic-ischemic encephalopathy

Jana Krystofova Mike, Yasmine White, Rachel S Hutchings, Christian Vento, Janica Ha, Anya Gunewardena, Hadiya Manzoor, Donald Lee, Courtney Losser, Kimberly Arellano, Oona Vanhatalo, Elena Seifert, Bo Wen, Lu Wang, Aijun Wang, Brian D Goudy, Payam Vali, Satyan Lakshminrusimha, Jogarao VS Gobburu, Janel Long-Boyle, Yvonne W Wu, Jeffrey R Fineman, Donna M Ferriero, Emin Maltepe

- **Supplemental Methods**
- **Supplemental Figures**
- **Supplemental Tables**

Supplemental Methods

Animals and Randomization

White Dorper sheep of both sexes were used. HIE was induced via UCO in near term lambs at 141-143 days gestation (term ~ 147-150 days). In the randomized, blinded efficacy study, pregnant ewes and their lambs were randomized to either AZM or placebo treatment in a 2:1 allocation. Randomization was performed at the level of the ewe. Animals in the placebo arm received an equal volume of normal saline IV infusion at matched time points (Figure 1A). Study drug was prepared by an individual separate from the research team to allow the research team to remain blinded to treatment assignment. All animals in the randomized, blinded efficacy study as well as an additional historical group of uninjured control animals (n=10, did not undergo UCO), were exposed to the same instrumentation. Additional placebo was pooled from other studies, n= 18-31 to increase the sample size for assessment of the immunomodulatory and histological effects of AZM. Control n=4-21 was added to include a non-UCO comparison.

Neonatal hypoxia-ischemia

Time-dated pregnant ewes were fasted for 12-24 hours prior to surgery. The ewes were induced with ketamine and propofol and anesthetized with isoflurane for surgery according to IACUC approved Standard Operating Procedure, SC-20-112, "Sheep Anesthesia: Surgical Research Facility, H-Building at TRACS". Briefly, a jugular catheter or peripheral venous catheter was placed, and the ewe given 4 mg/kg slow push IV propofol, and 1-5mg/kg Ketamine. After anesthetic induction and intubation, the ventral abdomen was shaved and cleaned. Immediately prior to surgery, the pregnant ewes underwent ultrasound imaging under general anesthesia to confirm pregnancy. After ultrasound, the ewe was then transferred into the operating room where she is placed on a mechanical ventilator. Anesthesia was maintained with 1-5 % isoflurane through the endotracheal tube. The ventral abdomen was then given a standard surgical scrub (using either Betadine or Chlorhexidine and alcohol) and the ewe placed on maintenance intravenous (IV) fluids, usually 5-15 ml/kg/hr. Oxygenation of the ewe was monitored with an O_2 saturation probe and hemodynamics were monitored with a noninvasive blood pressure cuff. A midline incision along the ventral abdomen (6-10") was made and the uterus exposed. The ewe was given IV antibiotics (penicillin G potassium 10,000-20,000 units/kg and gentamicin 1-2 mg/kg). After exteriorization of the fetal head, the fetus was intubated with an appropriately sized cuffed endotracheal tube (ETT). The lung liquid was passively drained by gravity and the ETT was plugged to prevent gas exchange during gasping. Venous and arterial catheters were placed in the jugular vein and carotid artery for hemodynamic monitoring, blood sampling and drug administration. Asphyxia was induced by UCO until the onset of asystole. The umbilical cord was cut, lamb was delivered to a radiant warmer and following 5 minutes of asystole as assessed by invasive hemodynamic monitoring the lambs were resuscitated with positive pressure ventilation with a fraction of inspired oxygen FiO_2 of 1.0. Resuscitation was not initiated with room air as asystole and the need for chest compressions is universal given the severity of the model and therefore oxygen therapy is clinically indicated. After 30 seconds of ventilation, external chest compressions were initiated. Chest compressions continued for 60 second intervals before reassessing the heart rate and these efforts continued

for up to 15 minutes. Epinephrine (0.01 mg/kg) was administered intravenously if inadequate response to oxygen, ventilation and chest compressions was noted after 60 seconds of asystole following initiation of ventilation. Additional doses of epinephrine were given if animals were unresponsive to initial doses. Volume boluses were not provided as oxygen, epinephrine and chest compressions were typically sufficient to restore adequate perfusion following ROSC. ROSC was defined as sustained heart rate of 100 bpm (beats per minute) with SBP > 20 mmHg. During, as well as following resuscitation, a ventilator provided ongoing mechanical ventilation. Assisted ventilation was weaned and then discontinued when the lamb was spontaneously breathing > 50 % of the time and maintained a peripheral oxygen saturation > 85 % at an FiO_2 of 0.21. No intravenous fluids were administered during or after resuscitation. After extubation, the lambs were fed 2 oz every 4 hours by tube the first day. Afterwards, the lambs got fed 2-6 oz by bottle depending on size of lamb 4 times a day. If they were not able to bottle feed, they continued with tube feeding 2-4 oz 4 times a day with bottle attempts every day until they were euthanized. The lambs were assessed over a 6-day period to determine neurodevelopmental outcomes and euthanized on day 6 with an overdose of euthanasia solution (100 mg/kg pentobarbitone sodium, Lethabarb^{TM}, Virbac Pty. Ltd., Peakhurst, NSW, Australia).

Inclusion and exclusion criteria
Animals were allocated to experimental groups and included in the final statistical analysis following predefined inclusion and exclusion criteria. A successful UCO occlusion was confirmed during surgery using invasive hemodynamic monitoring and pulse oximetry, along with blood gas analysis. Only animals with asystole lasting > 5 min as assessed by invasive hemodynamic monitoring were included in the experiment. Death that occurred after ROSC was accounted for in neurological outcomes analyses.

Drug treatment
AZM was purchased from Covertus, Inc (Portland, ME). In the pilot pharmacokinetic (PK) dose-finding study, ewes received 1-2 g IV AZM over one hours starting one hour prior to cesarian section. Following resuscitation, lambs received 30 mg/kg intravenous (IV) AZM infused over one hour. Plasma PK samples were collected from each ewe immediately prior to and at the completion of AZM infusion. PK samples were collected from each lamb prior to UCO (baseline, prior to AZM infusion, pre-UCO) and at 4, 8, 24, 48, 72, 96, 120, and 144 hours following AZM treatment. Brain, liver, lung, and spleen samples were collected on day of life six for AZM quantification from a second cohort of lambs that received a lower dose of AZM (10 mg/kg IV AZM following delivery and 5 mg/kg enteral AZM at 24 and 48 hours of life). In the randomized efficacy study, treatment consisted of 2 g IV AZM administered over one hour to the ewe prior to delivery and 30 mg/kg IV AZM administered to the lamb as a one-hour IV infusion starting ten minutes after onset of resuscitation followed by two additional 15 mg/kg IV AZM doses at 24 and 48 hours of life. Animals in the placebo arm received an equal volume of normal saline IV infusion at matched time points.

Pharmacokinetic analysis
Plasma levels of AZM were quantified by a validated using liquid chromatography with tandem mass spectrometry (LC-MS/MS): To determine the AZM concentration in lamb plasma samples, 160 µL of acetonitrile containing 25 nM internal standard and 20 µL of acetonitrile was added into 20 µL of plasma samples. The mixture was vortexed for 10 min and centrifuged at 3500 rpm for 10 min. The supernatant was transferred to autosampler vials for LC–MS/MS analysis. Tissue samples were homogenized (Precellys tissue homogenizer, Bertin Technologies, Montigny-le-Bretonneux, France) with the addition of 20 % acetonitrile in water, with a ratio of 5:1 volume (mL) to weight of tissue (g). Since no blank lamb tissues were available, the tissue homogenization was diluted 10 times with blank plasma, and then treated using the same procedure as that for the plasma samples to extract the compound for LC–MS/MS analysis. Blank plasma, the samples from un-treated control groups, were used to exclude contamination and interference. The AZM analytical curve was constructed with 10 nonzero standards spiked in blank plasma by plotting the peak area ratio of AZM to the internal standard versus the sample concentration. The concentration range evaluated was from 1 to 1000 ng/mL in lamb plasma. Lamb plasma AZM concentrations (ng/mL) were determined by the LC–MS/MS method developed and validated for this study. The LC–MS/MS method consisted of a Shimadzu LC-20AD HPLC system (Kyoto, Japan), and chromatographic separation of the tested compound was achieved using a Waters XBridage reverse phase C18 column (5 cm × 2.1 mm internal diameter, packed with 3.5 µm) at 25 °C. Five microliters of the supernatant was injected. The flow rate of gradient elution was 0.4 mL/min with mobile phase A (0.1 % formic acid in purified deionized water) and mobile phase B (0.1 % formic acid in acetonitrile). An AB Sciex Qtrap 4500 mass spectrometer equipped with an electrospray ionization source (ABI-Sciex, Toronto, Canada) in the positive-ion multiple reaction monitoring mode was used for detection. Protonated molecular ions and the respective ion products were monitored at the transitions of m/z 749.5 > 591.4 for AZM and 455.2 > 425.2 for the internal standard. We adjusted the instrument settings to maximize analytical sensitivity and specificity of detection. Data was processed with the software Analyst (v 1.6, AB SCIEX, Concord, ON, Canada).

Percentage placental transfer was calculated at each plasma PK collection time point by the following equation:

$$\% \text{ Drug Transfer} = [\text{Concentration}_{(lamb)}/\text{Concentration}_{(ewe)}] * 100$$

PK parameters for AZM were estimated utilizing standard non-compartmental analysis with R package ‘NonCompart’ v0.6.0 in R v4.2.0 (Bae 2022) and with PumasAI/NCA.jl v1.2.10 (Pumas-AI Inc, Baltimore, MD, USA) followed by non-linear mixed effect modeling using Pumas v1.1 (Pumas-AI Inc, Baltimore, MD, USA). First, standard noncompartmental analysis was performed to provide initial estimates for PK parameters of interest including peak concentration (Cmax), half-life, and area under the concentration-time curve from dosing time to last measurement time ($AUC_{0\text{-}t}$) and area under the concentration-time curve from dosing time to infinity ($AUC_{0\text{-}\infty}$). In the non-linear mixed effects modeling, one- and two-compartment PK models were compared. The final model was parameterized in terms of clearance, intercompartmental clearance, volume of the peripheral compartment, and volume of the central compartment. To

model between-subject variability, all PK parameters were assumed to be lognormally distributed. Additive, proportional, and combined models for residual error were tested. Final model selection was based on the value of the log-likelihood and Akaike's Information Criterion estimates as well as visual inspection of standard goodness-of-fit plots and visual predictive check.

Azithromycin Quantification

Bioanalytical study using Liquid Chromatography with tandem mass spectrometry (LC-MS/MS): To determine the AZM concentration in lamb plasma samples, 160 µL of acetonitrile containing 25 nM internal standard and 20 µL of acetonitrile was added into 20 µL of plasma samples. The mixture was vortexed for 10 min and centrifuged at 3500 rpm for 10 min. The supernatant was transferred to autosampler vials for LC–MS/MS analysis. Tissue samples were homogenized (Precellys tissue homogenizer, Bertin Technologies, Montigny-le-Bretonneux, France) with the addition of 20 % acetonitrile in water, with a ratio of 5:1 volume (mL) to weight of tissue (g). Since no blank lamb tissues were available, the tissue homogenization was diluted 10 times with blank plasma, and then treated using the same procedure as that for the plasma samples to extract the compound for LC–MS/MS analysis. Blank plasma, the samples from untreated control groups, were used to exclude contamination and interference. The AZM analytical curve was constructed with 10 nonzero standards spiked in blank plasma by plotting the peak area ratio of AZM to the internal standard versus the sample concentration. The concentration range evaluated was from 1 to 1000 ng/mL in lamb plasma. Lamb plasma AZM concentrations (ng/mL) were determined by the LC–MS/MS method developed and validated for this study. The LC–MS/MS method consisted of a Shimadzu LC-20AD HPLC system (Kyoto, Japan), and chromatographic separation of the tested compound was achieved using a Waters XBridage reverse phase C18 column (5 cm × 2.1 mm internal diameter, packed with 3.5 µm) at 25 °C. Five microliters of the supernatant was injected. The flow rate of gradient elution was 0.4 mL/min with mobile phase A (0.1 % formic acid in purified deionized water) and mobile phase B (0.1 % formic acid in acetonitrile). An AB Sciex Qtrap 4500 mass spectrometer equipped with an electrospray ionization source (ABI-Sciex, Toronto, Canada) in the positive-ion multiple reaction monitoring mode was used for detection. Protonated molecular ions and the respective ion products were monitored at the transitions of m/z 749.5 > 591.4 for AZM and 455.2 > 425.2 for the internal standard. We adjusted the instrument settings to maximize analytical sensitivity and specificity of detection. Data was processed with the software Analyst (v 1.6, AB SCIEX, Concord, ON, Canada).

Placental Transfer

Percentage placental transfer was calculated at each plasma pK collection time point by the following equation:

$$\% \text{ Drug Transfer} = [\text{Concentration}_{(lamb)}/\text{Concentration}_{(ewe)}] * 100$$

PK parameter estimation

Standard noncompartmental analysis was performed with R package 'NonCompart' v0.6.0 in R v4.2.0 (Bae 2022) and with PumasAI/NCA.jl v1.2.10 (Pumas-AI Inc, Baltimore, MD, USA) to provide initial estimates for PK parameters of interest including peak concentration (Cmax),

half-life, and area under the concentration-time curve from dosing time to last measurement time ($AUC_{0\text{-}t}$) and area under the concentration-time curve from dosing time to infinity ($AUC_{0\text{-}\infty}$). In the non-linear mixed effects model developed using Pumas v1.1 (Pumas-AI Inc, Baltimore, MD, USA), one- and two-compartment PK models were compared. The final model was parameterized in terms of clearance, intercompartmental clearance, volume of the peripheral compartment, and volume of the central compartment. To model between-subject variability, all PK parameters were assumed to be lognormally distributed. Additive, proportional, and combined models for residual error were tested. Final model selection was based on the value of the log-likelihood and Akaike's Information Criterion estimates as well as visual inspection of standard goodness-of-fit plots and visual predictive check.

Neurobehavioral outcomes

We assessed the time (days) taken to reach normal lamb behavioral milestones after birth (head lift and shake; use of front and hind limbs; use of four legs; standing; walking) for a total score of 4 (Tab. S1). Ability to feed and activity at rest were evaluated separately and were reported as a sum score of 2. The severity of impairment was assessed based on the composite score of a motor function and feeding, activity.

Immunohistochemistry

Following euthanasia on day 6, brains were flushed with 500 mL of phosphate-buffered saline (PBS) and perfused with 500 mL of 4 % paraformaldehyde in 0.1 M phosphate buffer (pH 7.4). Brains were post-fixed in 4 % paraformaldehyde overnight and transferred to 20 % sucrose for 2 days and 30 % sucrose till they sank (14 days). Brains were then flash frozen in 2-methyl butane on dry ice and stored at − 80 °C. Coronal sections were cut on a cryostat (12 µm-thick serial sections). Double immunofluorescence labeling was performed on brain sections that were defrosted and air dried at room temperature for 1 h. Following antigen retrieval in 10 mM citrate buffer (pH 6.0) for 10 min at 80 °C and a PBS wash, sections were incubated in blocking solution (5 % normal donkey serum, 0.4 % Triton X-100 in PBS) for 1 h at room temperature (RT). Primary antibody incubation was done overnight at 4 °C with rabbit anti-GFAP (GFAP, 1:500, Z0334, Agilent); mouse anti-NeuN for neurons (NeuN, 1:200, MAB377, Millipore Sigma), goat anti-Iba1 for microglia (Iba-1, 1:200, NB100-1028, Novus Biologicals), mouse anti-caspase-3 (casp-3, 1:200, NB600-1235, Novus Biologicals), goat anti-oligodendrocyte transcription factor 2 for oligodendrocytes (Olig-2,1:200 AF2418, Novus Biologicals), rat anti-myelin basic protein (MBP, 1:200, NB600-717, Novus Biologicals) and mouse anti-adenomatous polyposis coli protein clone CC-1 (CC-1, 1:100, OP800100UG, Millipore Sigma). After three 5-min PBS washes, sections were incubated for 1 h at RT with appropriate secondary antibodies: donkey anti-goat Alexa Fluor 647 (1:500, A21447, Thermo Fisher), donkey anti-mouse Alexa Fluor 568 (1:500, A10037, Thermo Fisher), donkey anti-rat Alexa Fluor 594 (1:500, A-21209, Thermofischer) and donkey anti-rabbit Alexa Fluor 488 (1:500, A21206, Thermo Fisher). For nuclear staining, sections were stained with 4′,6-diamino-2-phenylindol for 5 min. Slides were then washed and coverslipped with ProLong Gold antifade (P36930, Invitrogen).

Image analysis

To define the anatomical localization of the injury, we grossly evaluated all areas of the brain on sections corresponding to s.640, and 1200 of Sheep Brain Atlas[45]at 5X magnification. For white matter injury analysis, we assessed periventricular white matter (PVWM), subcortical white matter of the cingulate and first parasagittal gyrus (SCWM1 and SCWM2). For the gray matter injury analysis, we evaluated histological changes in cortex of the cingulate and first parasagittal gyrus (Ctx1 and Ctx2), caudate (Caud), putamen (Put) and hippocampal areas of Ca1/2 and Ca3. For the final analysis, we acquired 3 confocal-like Z-stacks from the same anatomical area (25X oil objective, 10 µm thick, 1 µm Z step) using a Zeiss microscope equipped with the confocal-like optigrid device and Volocity software (version 6.3, Improvision, Perkin Elmer, Waltham, MA, USA). Every brain had a control with no primary antibodies for staining. Image capturing (using Volocity software) and analysis using Imaris software (version 9.6.2. Oxford Instruments America Inc., Pleasanton, CA) to assess NeuN, Olig-2, CC-1 and cleaved caspase-3 cells cell counts and Iba-1, GFAP, MBP volumes. Ca1/2, Ca3 NeuN- positive cells and cleaved caspase-3 cells were manually counted. The analysis was done in a blinded manner. We measured the number of cells that express NeuN, Cleaved caspase-3, Olig-2, CC-1 and total volume of cell bodies and fibers expressing Iba-1, GFAP, MBP per field of view measuring 1350 x 1050 x 10 μm^3 (1.4×10^7 μm^3).

Biochemical markers of inflammation

To evaluate the impact of AZM treatment on systemic inflammation, we measured the cytokine levels at 6 days after the UCO using multiplex bead assay (Milliplex Ovine Cytokine/Chemokine Panel, SCYT1-91K, Millipore Sigma). 25 µL of undiluted serum samples were centrifuged at 14 000 rpm for 10 min at 4 °C. The supernatant was extracted and mixed with assay buffer and premixed beads and incubated on a plate overnight at 4 °C with agitation. After washing, the detection antibodies were added to the plate, followed by 1 h incubation at the RT. Plate was washed and analyzed on Luminex 200™ (Millipore Sigma, Merck KGaA, Darmstadt, Germany). The output data were analyzed using Belysa™ software (version 1.1.0, Millipore Sigma, Merck KgaA, Darmstadt, Germany). We further collected complete blood count prior to the UCO (BSN), at 8h, and on days 1, 2, 3, 5 and 6. We assessed the differences in white blood cells (WBC), neutrophils (absolute neutrophil count, ANC), lymphocytes (absolute lymphocyte count, ALC), platelets (PLT), monocytes (Mono), eosinophils (Eos). We calculated system inflammation response index (SIRI)= ANC x (Mono/ALC), systemic immune inflammation index (SII)= PLT x (ANC/ALC), and ratios of neutrophils/lymphocytes (NLR= ANC/ALC), platelets/lymphocytes (PLR= PLT/ALC) and lymphocytes/monocytes (LMR= ALC/Mono).

Detailed Statistical Results

Power analysis, sample size calculation, attrition rate, blinding, and randomization

The sample size was set at 15 lambs per treatment arm, corresponding to 80 % power at detecting an 80 % relative risk reduction (RRR) in a test of two, ewe-clustered (intracluster correlation = 0.1) binomial proportions with 65 % control event and a 5 % type-I error rate (one-sided), assuming each ewe gives birth to either one or two lambs with equal probabilities.

These numbers already account for the type-II error rate inflation resulting from futility testing after the first seven outcomes from each group have become available and assume 15 % attrition rate. Early stopping for futility will happen if the p-value at the interim analysis exceeds the value of 0.2822. Researchers performing experiments and analyzing data were blinded to groups. Randomization using an envelope system was applied. Envelopes were prepared and allocated in a standardized and consistent manner to avoid any potential bias or errors in the allocation process. Envelopes were prepared for each ewe in advance, each containing a slip of paper indicating which treatment group the ewe will be assigned to. The envelopes were opaque and sealed to ensure that the allocation is concealed until the moment of randomization to prevent any potential bias or influence from the participant in the allocation process. During the randomization process, the allocator of treatment selected an envelope at random, and the contents of the envelope indicated which treatment group the ewe will be assigned to. Once the ewe has been allocated to a treatment group, the allocator of treatment recorded the allocation. The study is a double-blind, as the researchers performing experiments and animal care, and data analysts are all unaware of the treatment group assignments. The researchers performing all the experiments, including the injury, post-injury care, biochemical, histological and neurological outcomes analysis were blinded to the group assignment until all measurements have been collected. The data was separated into two groups, data analysis was performed and the identity of the groups was revealed after the analysis was performed.

Statistical analysis

Analyses of biochemical, hemodynamics and histological data were performed using Prism 9 (version 9.4.1, GraphPad Software, San Diego, CA). All data are shown as mean ± standard error of measurement. Differences were considered significant at $p < 0.05$. Data was subjected to a normality test. If the data passed normality test, the differences between two groups were assessed by t-tests, otherwise we applied Mann-Whitney test. Grouped data were analyzed using one-way and two-way analysis of variance and subsequently subjected to Smidak post hoc analyses. The data that did not pass the normality test were analyzed using Kruskal-Wallis test. The hemodynamic data was analyzed using grouped analysis of the individual group's means for a specific time point.

To analyze the effect of AZM on the ordinal Severity Score, proportional odds ordinal regression models[46] were fitted at individual time points. The control group consisted of the concurrent placebo arm. To incorporate fatalities in the analysis, lambs were assigned a score of '0' at the time of death and were censored thereafter. An identical approach was used for the Motor Score outcome, the only difference being a '-1' score being assigned to dead lambs. The treatment effects and confidence intervals reported for both these outcomes are in the log-odds-ratio scale. For the numeric Total Feeds + Activity outcome, after assignment of '-0.25' to dead lambs, a linear model for the square root-transformed outcome (with appropriate offset to ensure positivity) proved a good fit through residual diagnostics, and the reported results were subsequently the mean difference in that scale.

Due to the relatively small samples, p-values and uncertainty bounds were based on 10,000 bootstrap samples[47]rather than asymptotic. Comparisons were made between AZM-treated (AZM) and untreated placebo groups (Placebo).

Supplemental Tables

Table S1: Neurobehavioral assessment score:

Function	Neurological Milestone	Score
**Motor function**	Walking	4
	Standing	3
	Four limbs	2
	Front/hind limbs	1
	No movement/Spastic	0
**Feeding**	Nurses normally	1
	Suckling well once finds the bottle	0.5
	Requires assistance to find bottle; a few good suckles	0.25
	Minimal suckle, tube fed	0
**Activity at rest**	Lifts the head up, alert active	1
	Wakes up with stimulation, attempts to lift the head	0.5
	Sleepy; no head lift with stimulation	0

The severity of impairment was classified based on a composite score of motor function, feeding and activity. The highest score represents no impairment of the selected neurological function.

Table S2. Pharmacokinetic parameters of azithromycin in neonatal lambs

Parameter	Estimate [95% CI]	Between-Subject Variability (% CV) [95 % CI]
**Clearance (L/hr/3kg)**	**0.91 [0.68, 1.11]**	**39.6 [22.1, 52.9]**
**Intercompartmental Clearance (L/hr/3kg)**	**1.38 [0.79, 1.99]**	**82.7 [44.7, 111.4]**
**Volume of the peripheral Compartment (L/3kg)**	**28.3 [19.9, 40.9]**	**—**
**Volume of the Central Compartment (L/3kg)**	**2.67 [1.81, 4.24]**	**—**
**Proportional residual error (%)**	**29.0 [22.8, 34.7]**	**—**

CI-confidence interval, CV-coefficient of variation

A population PK model was developed using AZM concentrations from 94 plasma samples from 19 different lambs who received doses of 10-30 mg IV AZM. The range of observed AZM concentrations was 0.0360 mg/L to 18.45 mg/L. No samples were below the level of quantification (0.001 mg/L). One outlier sample from lamb #2 at the four-hour time point was removed prior to modelling as it fell outside the acceptable pre-determined statistical criteria for sample variance. Initial modeling steps showed that a two-compartment model with weight as a covariate was a better fit than a one-compartment model (D-2LL of -177, $p < 0.0001$ compared to one-compartment model). Between-subject variability (BSV) was removed from the volume of the central compartment and peripheral compartment, as very high shrinkage was observed, indicating an inability to identify

BSV in these parameters. A proportional residual error model resulted in a lower AIC compared to an additive or combined additive and proportional error model (34.5 vs 208.5 and 35.3, respectively).

Table S3: Biochemical data parameters

1A	preUCO			CPR		10 min			20 min			30 min			60 min		
	AZM	Placebo	Control	AZM	Placebo	AZM	Placebo	Control	AZM	Placebo	Control	AZM	Placebo	Control	AZM	Placebo	Control
pH	7.19±0.01	7.22±0.01	7.25±0.01	6.84±0.01	6.89±0.01	7.06±0.03	6.98+0.02	7.26±0.05	7.18±0.02	7.1±0.03	7.18±0.03	7.1±0.02	7.16±0.03	7.14±0.03	7.12±0.02	7.18±0.03	7.19±0.03
pO2 [mmHg]	17.18±1.08	18.58±1.64	21.83±7.48	0.84±0.19	0.78±1.77	248.3±35.46	253.6±43.48	51.36±18.96	129.7±24.16	144.3±35.94	45.13±7.00	108.7±21.22	123.4±30.85	46.00±10.88	53.28±8.42	55.79±8.32	38.67±5.92
pCO2 [mmHg]	70±2.4	65±2.01	60.43±1.49	134.9±2.72	126.5±3.32	58.88±5.2	73.24±8.85	54.00±5.58	41.19±2.44	55.83±5.66	64.29±6.33	43.23±2.11	48.36±3.05	65±4.21	56.48±2.3	53.23±2.52	60.40±5.38
BE [mmol/L]	-1.6±0.54	-0.63±1.01	0.08±1.12	-10.83±0.43	-9.2±0.78	-14.7±0.49	-14.8±0.74	-2.2±1.61	-13.3±0.63	-12.8±0.87	-4.03±1.51	-12.8±0.57	-11.4±0.89	-4.9±1.23	-10.39±0.99	-6.09±1.78	-4.75±1.61
Lac [mmol/L]	3.47±0.35	4.15±0.72	2.12±0.34	8.68±0.32	8.53±0.44	10.43±0.6	10.43±0.6	2.29±0.66	8.58±0.42	9.36±0.55	2.82±0.84	8.14±0.4	8.71±0.57	2.60±0.69	7.14±0.5	6.96±2.2	2.76±0.733
Glc [mg/dL]	17.06±0.93	15.82±1.55	14.25±2.41	00±00	00±00	66.86±7.4	46.09±7.32	15.29±5.69	62.50±6.31	57.18±6.38	40.75±6.65	68.62±8.15	58.55±3.76	58.25±8.16	59.05±7.41	32.36±4.31	44.67±8.55

1B	preUCO		60min		480min		Day 1		Day 2		Day 3		Day 5		Day 6	
	AZM	Placebo	AZM	Placebo	AZM	Placebo	AZM	Placebo	AZM	Placebo	AZM	Placebo	AZM	Placebo	AZM	Placebo
BUN [mg/dL]	5.79±1.83	4.48±2.24	6.44±2.06	6.99±4.67	16.01±5.59	7.20±4.28	27.92±10.31	8.41±4.15	33.72±14.21	7.96±3.66	20.90±11.85	7.98±3.55	18.68±9.34	2.38±0.56	17.92±7.7	3.289±0.43
Cr [mg/dL]	2.54±0.15	2.33±0.09	2.46±0.15	2.28±0.08	1.85±0.11	1.97±0.1	1.05±0.06	1.15±0.08	0.57±0.02	0.63±0.02	0.53±0.03	0.57±0.03	0.58±0.03	0.6±0.04	0.59±0.03	0.57±0.03
AST [U/L]	18.40±2.16	15.73±1.32	27.04±4.22	30.49±2.5	52.17±6.33	41.68±4.84	76.08±6.96	58.87±4.84	77.34±10.93	61.53±5.12	75.36±9.56	59.18±5.13	61.96±4.33	54.70±6.31	61.70±4.53	52.88±3.45
ALT [U/L]	5.79±1.83	4.48±2.24	6.44±2.06	6.99±4.67	16.01±5.59	7.20±4.28	26.49±5.76	8.41±4.15	33.74±14.2	7.96±3.66	22.11±12.6	7.98±3.55	18.68±9.34	2.38±0.56	17.92±7.73	3.29±0.43

Significance is as follows: ⇔ *p<0.05,* ⇔ *p<0.01,* ⇔ *p<0.001,* ⇔ *p<0.0001*

1A, UCO leads to profound acidosis in both AZM-treated group, as well as placebo compared to controls (Controls data was used with permission from Mike et al.[23]). The AZM and Placebo groups did not differ in oxygen, carbon dioxide levels, base excess and level of hyperlactemia. Both, AZM and Placebo experienced hyperoxia and hyperglycemia as a result of stress oxygen use during the CPR (hyperoxia) and stress response to the UCO (hyperglycemia). **1B,** no differences in end organ function markers were noted between the AZM and Placebo groups. The data was analyzed using Mixed-effect analysis with Sidak's correction for multiple comparisons. The CPR data was analyzed using t-test or Mann-Whitney test as appropriate. Data in the table are mean ± SEM. For 1A: AZM: n=8-21, Placebo: n=13, Control: n=8; for 1B: AZM:9-18, Placebo:6-11. *preUCO-baseline pre-UCO; CPR-cardiopulmonary resuscitation; glc-glucose; BE-base excess; lac-lactate, BUN- blood urea nitrogen, Cr- creatinine, AST- aspartate aminotransferase, ALT- alanine transaminase. AZM- azithromycin-treated group.*

Supplemental Figures

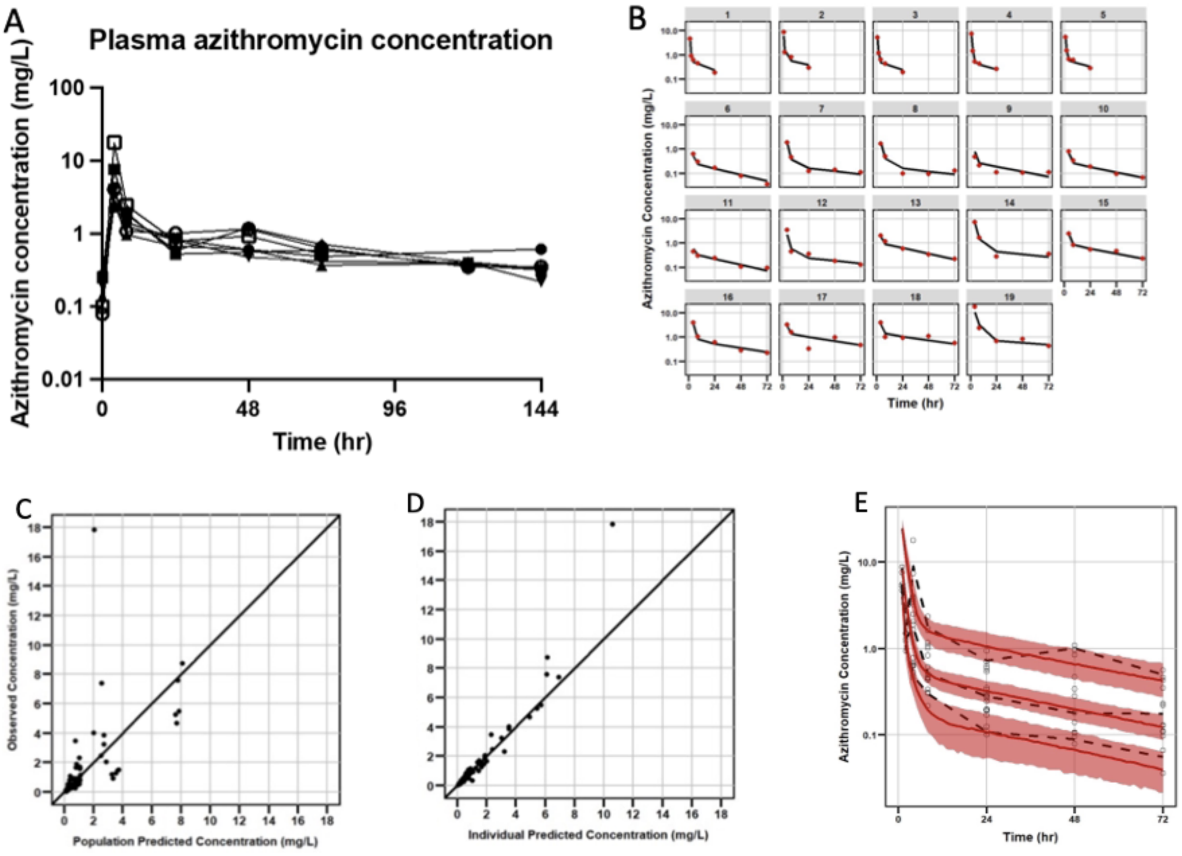

Figure S1. A, Plasma AZM concentration over time for lambs exposed to a 2g IV AZM dose to the ewe prior to delivery, 30 mg/kg IV AZM following resuscitation and two additional doses of 15 mg/kg IV AZM at 24 and 48 hours of life (n=7). **B,** Individual model prediction fits. Red diamonds represent observations, solid black line represents predicted AZM concentration. **C,** Goodness of fit plots for the final population pharmacokinetic model for population and **D,** individual predicted concentrations. Black circles represent individual observations, solid black line represents linen of identity. **E,** Visual predictive check of the final population pharmacokinetic model for 1000 simulated individuals. Open circles represent observations, dashed black line represent the 5th, 50th, and 95th percentile of the observations, solid red lines represent the 5th, 50th, and 95th percentile of the simulated data; red areas represent the 95% prediction intervals for the 5th, 50th, and 95th percentiles.

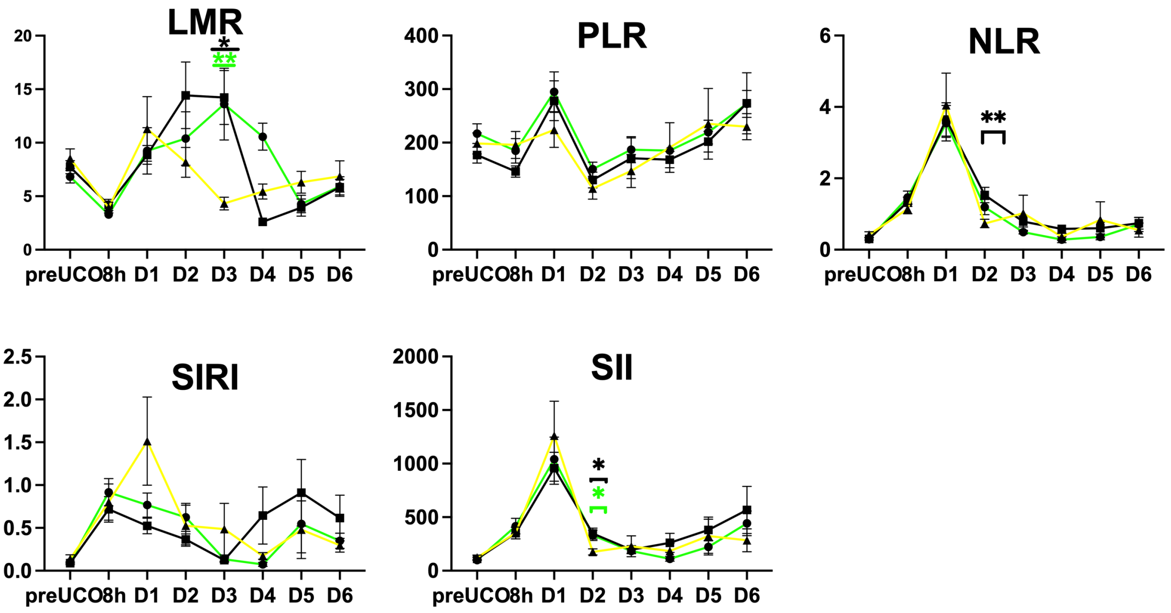

Figure S2: Peripheral markers of inflammation- inflammatory indices: Additional differences were noted in peripheral blood cell indices SII, LMR and NLR in AZM and placebo lambs compared to controls (Controls data was used with permission from Mike et al.[23]). The ratios were evaluated using Mixed-effect analysis with Sidak correction for multiple comparisons. Data are presented as mean ± SEM. Compared are AZM: n= 7-12, Placebo: n=7-21, Control: n=4-21. *SIRI- systemic inflammation response index (SIRI= absolute neutrophil count*Monocytes/ absolute lymphocyte count), SII- systemic immune-inflammation index (SII= absolute neutrophil count*Platelets/ absolute lymphocyte count), NLR- neutrophil to lymphocytes ratio, PLR: platelets to lymphocytes ratio, LMR- lymphocytes to monocytes ratio.* *p<0.05, **p<0.01.

The ARRIVE guidelines 2.0: author checklist

The ARRIVE Essential 10

These items are the basic minimum to include in a manuscript. Without this information, readers and reviewers cannot assess the reliability of the findings.

Item		Recommendation	Section/line number, or reason for not reporting
**Study design**	1	For each experiment, provide brief details of study design including:<br>a. The groups being compared, including control groups. If no control group has been used, the rationale should be stated.<br>b. The experimental unit (e.g. a single animal, litter, or cage of animals).	1a azithromycin-treated vs placebo vs controls<br>1b single animal
**Sample size**	2	a. Specify the exact number of experimental units allocated to each group, and the total number in each experiment. Also indicate the total number of animals used.<br>b. Explain how the sample size was decided. Provide details of any *a priori* sample size calculation, if done.	Total number and number in each group were described in Methods and Figure legends.<br>Sample size is defined in detail in Supplemental Material under Detailed Statistical Analysis
**Inclusion and exclusion criteria**	3	a. Describe any criteria used for including and excluding animals (or experimental units) during the experiment, and data points during the analysis. Specify if these criteria were established *a priori.* If no criteria were set, state this explicitly.<br>b. For each experimental group, report any animals, experimental units or data points not included in the analysis and explain why. If there were no exclusions, state so.<br>c. For each analysis, report the exact value of *n* in each experimental group.	3a Inclusion and exclusion criteria are defined in Supplemental Methods<br>3b Number of successful animals is reported in figure legends<br>3c exact number is described in the figure legends
**Randomisation**	4	a. State whether randomisation was used to allocate experimental units to control and treatment groups. If done, provide the method used to generate the randomisation sequence.<br>b. Describe the strategy used to minimise potential confounders such as the order of treatments and measurements, or animal/cage location. If confounders were not controlled, state this explicitly.	4a Lambs were randomly assigned to treatment vs placebo group. Investigators performing the experiments and data analysis were blinded to the experimental groups.<br>4bAnimals from different groups were housed in the same room to minimize the confounders prior and after the experiment. If applicable, the samples were processed in bulks under the same conditions.
**Blinding**	5	Describe who was aware of the group allocation at the different stages of the experiment (during the allocation, the conduct of the experiment, the outcome assessment, and the data analysis).	Investigators performing the experiments and analyzing data were blinded to experimental groups. Experimental key was held by an individual not involved in the experiment and was not shared until the data was analyzed.
**Outcome measures**	6	a. Clearly define all outcome measures assessed (e.g. cell death, molecular markers, or behavioural changes).<br>b. For hypothesis-testing studies, specify the primary outcome measure, i.e. the outcome measure that was used to determine the sample size.	All measurements are defined in the methods and results.<br>As described in Methods and Results, the primary outcomes measured were improvement in histological and neurological outcomes.

**Statistical methods**	7	a. Provide details of the statistical methods used for each analysis, including software used.<br>b. Describe any methods used to assess whether the data met the assumptions of the statistical approach, and what was done if the assumptions were not met.	7a,b Statistics and statistical approach to assumptions was described in Methods and Supplemental Material
**Experimental animals**	8	a. Provide species-appropriate details of the animals used, including species, strain and substrain, sex, age or developmental stage, and, if relevant, weight.<br>b. Provide further relevant information on the provenance of animals, health/immune status, genetic modification status, genotype, and any previous procedures.	Species, sex, age was described in Methods
**Experimental procedures**	9	For each experimental group, including controls, describe the procedures in enough detail to allow others to replicate them, including:<br>a. What was done, how it was done and what was used.<br>b. When and how often.<br>c. Where (including detail of any acclimatisation periods).<br>d. Why (provide rationale for procedures).	The procedures were described in detail in Methods and Supplemental Material
**Results**	10	For each experiment conducted, including independent replications, report:<br>a. Summary/descriptive statistics for each experimental group, with a measure of variability where applicable (e.g. mean and SD, or median and range).<br>b. If applicable, the effect size with a confidence interval.	All results and statistics are described in Results, under Figure legends and in Supplemental material in detail.
